# Characteristics of blunt and penetrating trauma among victims of physical violence: A retrospective study

**DOI:** 10.1186/s12889-024-18978-w

**Published:** 2024-07-31

**Authors:** Behzad Zohrevandi, Mahsa Farzaneh Shahrestani, Hamideh Mohammadnia, Kamran asadi, Naema Khodadadi-Hassankiadeh

**Affiliations:** 1 Guilan Road Trauma Research Center, Trauma Institute, Guilan University of Medical Sciences, Poursina Hospital, Namjoo St, Postal Code, Rasht, Guilan 4193713194 Iran; 2 School of Medicine, Guilan University of Medical Sciences, Rasht, Iran; 3 Determinants of Health Research Center, Trauma Institute, Guilan University of Medical Sciences, Rasht, Iran; 4 Orthopaedic Research Center, Department of Orthopaedic Surgery, School of Medicine, Poursina Hospital, Guilan University of Medical Sciences, Rasht, Iran

**Keywords:** Blunt, Penetrating, Trauma, Physical violence, Emergency service

## Abstract

**Background:**

A significant number of referrals to the emergency departments is due to violence and conflict leading to serious injuries and death. The increasing number of such cases highlights the urgent need for investigating victimization of physical violence.

**Aim:**

The purpose of this study was to determine the frequency of demographic and clinical characteristics in victims of violence and classify them based on penetrating or blunt trauma.

**Methods:**

The data of the patients who had been the victims of violence in 2020 were extracted from the Trauma Registry System(TRS) of the Guilan Road Trauma Research Center(GTRC). All analyses were performed using SPSS software version 24. The significance level was considered less than 0.05.

**Results:**

There was a significant difference in the type of violence-related trauma in different age groups (*P* < 0.001), based on sex (*P* = 0.002), and marital status (*P* = 0.012). A significant difference also existed between the trauma type and clinical variables including smoking (*P* = 0.032), history of alcohol drinking (*P* = 0.005), and other substance use (*P* = 0.002), the anatomical location of injury (*P* < 0.001) and therapeutic interventions (*p* < 0.001(.

**Conclusion:**

Most of the clients of the violence suffered from blunt trauma, the severity of which was mild, and they were treated supportively without the need for surgery. Blunt trauma was seen more in women, divorcees, over 40 years and non-smokers. Penetrating trauma was observed more in lower limb injuries and alcohol and amphetamine users. Prevention programs and educational interventions should be programmed for the society long before men alcohol and amphetamine abusers reach their fourth decade of life. Accurately recording the type of physical violence, and the weapon used, and determining the injury severity score in TRS can lead to more reliable results in researching the field of violence issues.

## Introduction

Trauma is the most pervasive cause of death in the fourth decade of life and the third cause of death in all age groups after cardiovascular diseases and cancer [1–3]. Each year, about 500,000 people are murdered around the world, which is increasing at an annual rate of 2% [4, 5].

According to a 2015 telephone survey conducted by the CDC )The Centers for Disease Control and Prevention(, approximately 47% of women and 47% of men experienced psychological aggression such as humiliating or controlling behavior. One in three women and one in seven men in the United States report experiencing severe physical violence from a sexual partner in their lifetime. Gun violence has become an epidemic in the US which is significantly higher than that of all high-income countries, causing more than 33,000 deaths and 79,000 non-fatal injuries each year [6, 7]. According to reports, penetrating trauma has been more than doubled in the last 6 years. It is the most common type of trauma caused by violence. Interpersonal violence is the most common etiology. The characteristics of the observed injury differ depending on the perpetrators and their motivations [8].

Violence is a critical problem in emergency departments. According to previous studies, older unemployed men [9, 10] with financial problems [11] and from ethnic minorities [12] having antisocial fathers/mothers with a history of child abuse have been more exposed to stressful life events, mental health problems, and repeated aggressive behaviors [13, 14]. In addition, perpetrators of violence were more likely to have substance and alcohol use [15], and history of legal problems, crime [15, 16], attempted suicide, and murder [12].

Female victims of violence were usually young, and unmarried, and belonged to low social class [15]. Another study found low education level, being divorced, and having one child from a previous relationship common among these women [10, 16]. Spousal violence against women is the most common cause of trauma in pregnancy [17, 18].

In one study, the majority of sexual violence victims reported that the perpetrator had used alcohol or substance. Moreover, 29.7% of women and 32.4% of men victims reported involuntary use of alcohol/substance while 84.0% of female and 82.6% of male victims reported voluntary use [19].

Violence and the subsequent penetrating trauma are common in those who consume amphetamines and present to the emergency room. They need immediate surgical intervention due to the high risk of mortality [20].

In a study in Tunisia, out of a total of 198 victims of violence, 6.8% had severe injuries. Severe physical violence was statistically related to the type of weapon used (sharp or thermal force), type of injury (head injury, sharp wound, penetrating injury, bruise, and fracture-dislocation), and location of injury (head and face) [21]. In a systematic review conducted by Iranian researchers, the prevalence of domestic violence has been estimated to be 66% in Iran and 62% only in the north of the country, which indicates the high prevalence of domestic violence and requires urgent interventions by legal authorities [22].

Few studies have inspected blunt and penetrating trauma caused by violence. They have only examined injuries to the neck [23], head and neck [8], and liver [24]. The distribution of penetrating and blunt trauma to the whole body after the occurrence of violence has not been scrutinized so far. Studying the cases exposed to violence can increase the awareness of related injuries in society and be a primary important ground for shaping new measure for better monitoring crimes. In fact, the findings of these studies can be very helpful in developing intervention programs to make society a safer place, as well as in reducing treatment costs related to violence, and saving manpower and time. Therefore, the present research was conducted with the aim of determining the frequency of demographic and clinical characteristics in the victims exposed to violence and classifying them based on penetrating or blunt trauma.

## Method

### Setting

Using a retrospective design, the data were obtained from the TRS of the GRTRC, Rasht, Iran after obtaining approval from the ethics committee of Guilan University of Medical Sciences (IR.GUMS.REC.1398.421). The ethics committee confirmed that due to the retrospective nature of the study, there is no need to obtain informed consent from the samples. The victims who had been exposed to violence and referred to Poursina Hospital, a key tertiary referral center in the north of Iran (Guilan Province) from April to March 2020 were investigated.

### Inclusion criteria

Injured people aged ≥ 15 years, whose type of trauma was recorded as violence in the TRS, were included in the research. Patients with incomplete or distorted registration information were excluded from the study.

### Data gathering

In this study, after obtaining the required ethical permissions, the researchers obtained the following information from at TRS of GRTRC. The variables under study included age, sex, marital status, arrival time (hour, day and season), history of smoking, alcohol drinking, opium, and other drug use (Hashish, marijuana, amphetamines, cocaine), type of trauma (penetrating/blunt), anatomical location of the injury (head/neck, chest, abdomen, lower and upper limbs), therapeutic (supportive treatment, suture and surgery( and the final outcome (death, discharge with recovery, discharge with personal consent or transfer to another center) were recorded in a pre-prepared checklist.

### Sample size

To determine the sample size, a formula was used to estimate its ratio in a society. Considering the ratio of 0.14 in a previous study [25], error level of 0.05 and d = 0.039, the minimum sample size of 305 was obtained.

$$[{{\rm{n}}_0} = \frac{{{{\left( {{{\rm{z}}_{1 - \frac{\alpha }{2}}}} \right)}^2} \cdot {\rm{p}}(1 - {\rm{p}})}}{{{{\rm{d}}^2}}} = \frac{{{{(1.96)}^2}0.14(1 - 0.14)}}{{{{(0.039)}^2}}} = 304.09 \cong 305]$$

Season was considered an important variable in this study, therefore, we considered a ratio for each season. First we obtained the total number of trauma cases caused by violence in 2020 and per season, then the number of cases in each season was divided by the total number of hospital records in 2020. The result was multiplied by 305 (calculated sample size) to randomly obtain the number of records we needed in each season.

### Data analysis

In order to analyze data, the Chi-square and the Fisher exact tests were used. Then pairwise comparisons were made using the Chi-square test with Bonferroni correction. All the analysis were performed using SPSS software version 24. The significance level was considered less than 0.05 in all tests.

## Results

The majority of victims (34.8%, *n* = 106) were in the age group of 20–29 (34.8%), 89.8% (*n* = 274) )age mean of 34.20 ± 12.09 years (were male and 55.1% (*n* = 168) were married. Most of the referrals to hospital were on non-holidays/working days (73.1%, *n* = 223), tuesday (26.56%, *n* = 81) in summer (31.5%, *n* = 96) and from 16 PM to 12 AM (48.2%, *n* = 147) (Table 1).

**Table 1:** Demographic characteristics of the victims of physical violence (*N* = 305)

Measure		*N* (%)
Male		274(89.8)
Female		30(10.2)
Age	15–19	21(6.9)
	20–29	106(34.8)
	30–39	91(29.8)
	≥ 40	87(28.5)
Marital status	Single	127(41.6)
	Married	168(55.1)
	Divorced	4(1.3)
	Widowed	6(2)
Referral day	Holiday	82(26.9)
	Non-holiday	223(73.1)
Referral season	Spring	84(27.5)
	Summer	96(31.5)
	Autumn	84(27.5)
	Winter	41(13.4)
Referral hours	00:00–08:00	91(29.8)
	08:00–16:00	67(22)
	16:00–00:00	147(48.2)
Week days	Satarday	23(7.54)
	Sunday	18(5.90)
	Monday	52(17.05)
	Tuesday	81(26.56)
	Wednsedy	49(16.06)
	Thursday	21(6.88)
	Friday	61(20)

The majority of the victims exposed to physical violence were non-smokers (54.1%, *n* = 165) and had no history of alcohol drinking (93.4%, *n* = 285), opium (86.2%, 263) and use of other drugs (97.7%, *n* = 298). More than half of the victims had blunt injuries (57%, *n* = 174). Less than half had received supportive care (42%, *n* = 128), for head/neck injury (43%, *n* = 131) and 83.6% (*n* = 255) were discharged with recovery (Table 2).

**Table 2:** Clinical characteristics of victims of physical violence (*N* = 305)

Measure	Categories	*N* (%)
Smoking history	Yes	140(45.9)
	No	165(54.1)
Alcohol drinking history	Yes	20(6.6)
	No	285(93.4)
Opium use history	Yes	42(13.8)
	No	263(86.2)
Amphetamine use history	Yes	7(2.3)
	No	298(97.7)
Trauma type	Penetrating	131(43)
	Blunt	174(57)
Anatomical location of injury	Head/neck	128(42)
	Chest	56(18.4)
	Abdomen/pelvis	45(14.8)
	Upper limbs	65(21.3)
	Lower limb	11(3.6)
Therapeutic intervention	Supportive treatment	131(43)
	Suture	130(42.6)
	Surgery	44(14.4)
Outcome	Recovery	255(83.6)
	Discharge with personal consent	40(13.1)
	Transfer to another hospital	10(3.3)
	Death	0(0)

The results of the analysis with the Chi-Square test exhibited that the mechanism of violence-related trauma significantly varied in different age groups (*P* < 0.001), so that blunt trauma was more common in people ≥ 40 years of age. There was a significant difference based on sex (*p* = 0.002) in the groups. A higher percentage of women had blunt trauma than men. There was a significant difference based on marital status (*P* = 0.012). Blunt trauma was observed more in divorcees than other groups. However, there was no significant difference between the mechanism of violence-related trauma with day (*P* = 0.281), season (*P* = 0.341), hospital arrival hour (*P* = 0.199) and week days(*P* = 0.971) (Table 3).

**Table 3:** Comparison of demographic characteristics of victims of physical violence according to trauma type

Measure	Blunt *N* (%)	Penetrating *N* (%)	*p*-value
Age group			
15–19	(42.9) 9	(1.57) 12	*0.000
20–29	(46.46) 49	(8.53) 57	
30–39	(8.53) 49	(0.246) 42	
≥ 40	(77) 67	(23) 20	
**Sex**			*0.002
Male	(54) 148	(46) 126	
Female	(83.9) 25	(1.16) 5	
**Marital status**			
Single	(5.46) 59	(5.53) 68	**0.012
Married	(7.63) 107	(0.336) 61	
Divorced	(83.3) 5	(0.716) 1	
Widowed	(75) 3	(25) 1	
**Referral day**			
Holiday	(0.251) 42	(848.) 40	*0.281
Non-holiday	(0.295) 132	(840.) 91	
**Referral season**			
Spring	84(27.5)	(50) 42	*0.341
Summer	96(31.5)	(8.43) 42	
Autumn	84(27.5)	(0.936) 31	
Winter	41(13.4)	(39) 16	
**Referral hours**			
00:00–08:00	(0.549) 45	(0.550) 46	*0.199
08:00–16:00	0.2)58) 39	0.8)41) 28	
16:00–00:00	(2(61. 90	0.8)38) 57	
**Week days**			
Satarday	12(6.9)	11(8.4)	*0.971
Sunday	9(5.2)	9(6.9)	
Monday	28(16.1)	24(18.3)	
Tuesday	48(27.6)	33(25.2)	
Wednsedy	30(17.2)	19(14.5)	
Thursday	12(6.9)	9(6.9)	
Friday	35(20.1)	26(19.8)	

*Pearson Chi-Square **Fisher’s Exact Test

Comparing the clinical characteristics with the mechanism of violence-related trauma using the Chi-Square test indicated a significant difference between the type of trauma and smoking (*p* = 0.032). A remarkable percentage of non-smokers had blunt trauma compared with smokers. morover, penetrating trauma was higher in alcohol consumers than in subjects without a history of alcohol drinking (*P* = 0.005). There was a significant difference between the type of trauma and the history of amphetamine use (*P* = 0.002); people who used amphetamine had more penetrating trauma compared to people without such a history.

Moreover, there was a significant difference between the groups based on the anatomical locations (*P* < 0.001). Patients with trauma in their lower limbs had more penetrating trauma compared to other body areas. A significant difference existed between the trauma type and the required therapeutic interventions (*p* < 0.001) so that most people with blunt trauma received more supportive treatment. However, no significant difference was observed between the trauma type and history of opium use (*P* = 0.307) and the hospital outcomes (*P* = 0.276) (Table 4).

**Table 4:** Comparing the clinical characteristics of victims of physical violence according to trauma type

Measure	Blunt *N* (%)	Penetrate *N* (%)	*p*-value
Smoke usage			
Yes	(7.50) 71	(3.49) 69	*0.032
No	(4.62) 103	(6.37) 62	
**Alcohol drinking history**			
Yes	(25) 59	(75) 15	*0.005
No	(3.59) 169	(7.40) 116	
**Opium use history**			
Yes	(3.64) 27	(7.35) 15	*0.307
No	(9.55) 147	(1.44) 116	
**Amphetamine use history**			**0.002
Yes	(0) 0	(100) 7	
No	(4.58) 174	(6.41) 124	
**Anatomical location**			
Head & neck	(4.84) 108	(15.6) 20	*0.000
Chest	(4.30) 17	(69.6) 39	
Abdomen & pelvis	(2.62) 28	(37.8) 17	
Upper limbs	(3.32) 21	(67. 7) 44	
Lower limbs	(0) 0	(100) 11	
**Therapeutic interventions**			
Supportive treatment	(4.92) 121	(7.6) 10	*0.000
Suture	(8.33) 44	(66.2) 86	
Major surgery	(5.20) 9	(79.5) 35	
**Outcomes**			
Recovery	(9.54) 140	(45.1) 115	*0.276
Discharge with personal consent	(5.67) 27	(32.5) 13	
Transfer to another hospital	(70) 7	(30) 3	
Death	0	0	

* Pearson’s Chi-Square ** Fisher’s Exact Test

Analyzing the two-by-two comparisons of head and neck injuries as well as other injuries revealed a significant difference between the groups in terms of trauma type. Penetrating trauma was less in the head and neck than other body parts. There was also a significant difference between the trauma type reported for chest and the abdominal-pelvic area. Blunt trauma was more in the latter than in the former. A significant difference manifested between the abdominal-pelvic area and the upper and lower limbs which had more penetrating trauma than the abdominal-pelvic area(Table 5).

**Table 5:** Pairwise comparisons between head-neck areas with other site of injuries

Site of injury	Head-neck	Chest	Abdomen-pelvis	Upper limb	Lower limb
Head/neck					
Chest	0.001				
Abdomen/pelvis	0.002	0.001			
Upper limb	0.001	0.188	0.002		
Lower limb	0.001	0.034	0.001	0.029	

## Discussion

In the present study, 57% of the victims of violence had blunt trauma. Similarly in a study, in most cases (41.6%) punches were the main cause of trauma, not sharp objects [26]. In another study, blunt trauma was more common than penetrating trauma in victims of intimate partner violence. In addition, in the present study, the majority of traumas were to the head/neck, upper limb, chest, abdomen, and lower limb, respectively. In research performed on victims of sexual partner violence, the most common injuries were to the head/face, followed by the chest (mostly rib fractures), upper extremity, and abdomen. Concurrent injuries to multiple body regions were common, especially craniofacial and upper limbs [27].

The findings of the present study illustrated that blunt trauma was more common in women exposed to violence than male victims. There was a statistically significant relationship between type of trauma and sex. Similarly, in a study, the majority of female victims of partner sexual violence suffered from blunt trauma to the upper body [28]. In another study that examined gender differences in cranial trauma in individuals exposed to violence, men were more injured than women and had more multiple wounds [29]. In another study, it was reported that 96.4% of penetrating trauma caused by gunshot was observed in men.

In the present study, older than 40 years were significantly more exposed to blunt trauma than other age groups. Similarly, in the study by Al-Koudmani and colleagues (2012) penetrating heart injuries occurred in the age group of 10 to 40 years old, of which 10 cases (8 cases of stab wounds and 2 cases of gunshot wounds) were repaired with simple surgery without the need for cardiopulmonary surgery(bypass) [30]. In a study, 90% of the sexual violence victims were women with mean age 37 years old [27]. In another study, both knife wounds and stab wounds had the highest incidence at the age of 20–25 years. There was no change in the age distribution of gunshot wounds in 2012–2018, but the age distribution of stab wounds showed a decreasing trend until 2018 [31].

In the present study, a significant relationship was noticed between marital status and type of trauma. This means that divorced people experiencing violence had more blunt trauma. Demographic factors such as young age, race/ethnicity, and marital status shape the risk of intimate partner violence victimization. The factors that cause the continuation of a woman’s relationship with a violent sexual partner are the lack of education, income and employment, in other words, financial dependence, in addition, the feeling of responsibility for children, both before and after divorce, can increase the risk of women becoming victims of violence [32].

In our study, non-smokers exposed to violence had significantly more blunt trauma than smokers.

Moreover a significant number of the exposed with a history of alcohol consumption had penetrating trauma compared to those without a history. Moreover, victims with a history amphetamin use reported more penetrating trauma than those without such a history. According to findings of a study on intimate partner violence, more than 50% of the victims had comorbid psychiatric disorders or substance use [27]. Kiani et al. in their study reported a history of drug use in 7.9% of victims, alcohol use in 2.5%, and a history of drug use in 1.8% of cases. However, they did not report the type of abused substance causing penetrating or blunt trauma [26]. There is strong evidence that alcohol consumption can exacerbate aggressive behavior and lead to domestic violence or serious crimes [33–36]. A systematic review found that neither amphetamine nor methamphetamine acutely increased aggression [37]. In another research, methamphetamine use was a risk factor for both violence perpetration and victimization in the general population [38]. It may not be possible to show scientific evidence to discuss these results, but in a Persian proverb they say that Halva(A kind of traditional sweet) is not spread in a fight. In other words, in violence, you should expect sharp damage, not soft damage. Especially if the parties to the fight are alcoholics and drug dependence. Future researchs should evaluate type of trauma in current smoker, alchol consumption and methamphetamine user.

In an analysis performed on the demographic characteristics of women exposed to violence, 43.1% of women were in the age group of 30 to 39 years. The face was the main injured area in 59 victims (16.9%). Fifty-five (15.7%) patients had upper limb injuries, followed by face and upper limb wounds recorded in 36 (10.2%) women. According to the statements of the victims, the main reasons for committing violence were the frequent use of alcohol and drugs [39].

In our scrutiny, there was a significant difference based on the location of the injury. Penetrating trauma was more common in victims with lower limb trauma compared to other sites. Mellouki et al. analyzed the cases of death following exposure to violence and reported chest and abdominal lesions as the most common injuries in victims (34%) [40]. In another study conducted on the bodies of the dead female victims, in terms of the instrument used, blows with blunt objects were the most common mechanism of violence (44.44%), followed by sharp instruments (39%) and firearms (17%) [40]. Although the research did not separately specify the anatomical location of the injury.

Although most of the injured with blunt trauma received supportive treatment and did not need surgery, no significant difference was observed in terms of their final outcome when comparing the two types of trauma. Similarly, in a cohort study of 20,285 patients, after adjusting for confounding variables, blunt neck injuries were associated with a reduced probability of death and a significant reduction in the length of hospital stay [23]. In one study, however, the number of the exposed to violence who underwent medical treatment was the same as the number who underwent surgery (37.8 versus 37.8) [41]. In another study, the shock index (SI) and injury severity score (ISS) were worse in the blunt group: intra-abdominal trauma was significantly higher in the penetrating than in the blunt group, especially in the hollow organs. Furthermore, 84% of patients with penetrating injury underwent laparotomy while only 33% of blunt injury cases underwent laparotomy. Mortality rates were similar in both group. Non-surgical management was more feasible for blunt injuries (two-thirds of injuries) than for penetrating injuries [24]. AT the end of In, violence injuries rarely required surgical intervention and the risk of spinal cord injury was low [42].

The present study was conducted on the recorded data of violence victims. Considering that Poursina Hospital in Rasht City is a trauma referral center for all the cities of Guilan province and most of the trauma cases from across the province are referred to this center, the results of this study can be generalized. Besides, the comparison of penetrating and blunt trauma in terms of demographic and clinical factors is unique.

### Limitations

One of the limitations of the study was that it was not known whether our client was a hitman or a hitman. Therefore, when we write that alcoholics suffered more penetrating trauma, the reader thinks that how an alcoholic person who is aggressive himself became a victim and suffered penetrating trauma, while most alcoholics are expected to cause penetrating trauma. Therefore, specifying the multiplier of the multiple of recording such information in the trauma registration system can provide a better understanding for the readers. In addition, the type of violence (family, partner, and street) and the type of weapon used are not recorded. Due to the fact that all the necessary items to determine the ISS were not registered in the trauma research center registration system, we encountered many obstacles to report whether our penetrating trauma was more severe or blunt trauma. Despite having two variables, the type of therapeutic intervention and the final outcome of the hospital, which indirectly reflect the severity of physical injuries, we still needed the ISS to complete the discussion and compare with the results of studies in other countries.

## Conclusion

Most of the clients of the violence suffered from blunt trauma, the severity of which was mild, and they were treated supportively without the need for surgery. Blunt trauma was seen more in women, divorcees, over 40 years and non-smokers. Penetrating trauma was observed more in lower limb injuries and alcohol and amphetamine users. Prevention programs and educational interventions to reduce the incidence of conflict and its severe injuries should be focused on alcohol and amphetamine abusers on men under 40 years of age. The low number of alcohol and drug use compared to other similar studies indicates that these data are underreported. It should be emphasized that the type of physical violence and ISS is not usually recorded in the TRS which inhibited the possibility of comparing the results of this study with other studies.

## Data Availability

All data generated or analysed during this study are included in this published article.
